# Clinical trial enrollment at a rural satellite hospital during COVID-19 pandemic

**DOI:** 10.1017/cts.2021.777

**Published:** 2021-04-08

**Authors:** Yub Raj Sedhai, Melissa Sears, Alessandra Vecchiè, Aldo Bonaventura, Joan Greer, Kathryn Spence, Hilary Tackett, Juanita Turner, Mary Pak, Nimesh Patel, Mellisa Black, George Wohlford, Rick Earle Clary, Christina Duke, Mary Hardin, Heather Kemp, Anna Priday, Earl Kenneth Sims, Virginia Mihalick, Ai-Chen Ho, Ikenna Ibe, Mary Harmon, Roshanak Markley, Benjamin Van Tassell, Antonio Abbate

**Affiliations:** 1 VCU Health, Community Memorial Hospital, South Hill, VA, USA; 2 VCU Health, Medical College of Virginia Hospital, Virginia Commonwealth University, Richmond, VA, USA

**Keywords:** COVID-19, SARS-CoV-2, canakinumab, clinical trial, telemedicine

## Abstract

**Introduction:**

Controlled clinical trials (CCTs) have traditionally been limited to urban academic clinical centers. Implementation of CCTs in rural setting is challenged by lack of resources, the inexperience of patient care team members in CCT conductance and workflow interruption, and global inexperience with remote data monitoring.

**Methods:**

We report our experience during the coronavirus disease 2019 (COVID-19) pandemic in activating through remote monitoring a multicenter clinical trial (the Study of Efficacy and Safety of Canakinumab Treatment for cytokine release syndrome (CRS) in Participants with COVID-19-induced Pneumonia [CAN-COVID] trial, ClinicalTrials.gov Identifier: NCT04362813) at a rural satellite hospital, the VCU Health Community Memorial Hospital (VCU-CMH) in South Hill, VA, that is part of the larger VCU Health network, with the lead institution being VCU Health Medical College of Virginia Hospital (VCU-MCV), Richmond, VA. We used the local resources at the facility and remote guidance and oversight from the VCU-MCV resources using a closed-loop communication network. Investigational pharmacy, pathology, and nursing were essential to operate the work in coordination with the lead institution.

**Results:**

Fifty-one patients with COVID-19 were enrolled from May to August 2020, 35 (69%) at VCU-MCV, and 16 (31%) at VCU-CMH. Among the patients enrolled at VCU-CMH, 37.5% were female, 62.5% Black, and had a median age of 60 (interquartile range 56–68) years.

**Conclusion:**

Local decentralization of this trial in our experience gave rural patients access to a novel treatment and also accelerated enrollment and more diverse participants’ representative of the target population.

## Introduction

Early after the emergence of coronavirus disease 2019 (COVID-19), it was evident that effective treatment options against infection from severe acute respiratory syndrome coronavirus 2 (SARS-CoV-2), that is, the virus, and COVID-19, that is, the disease caused by the virus, were greatly needed [1,2]. The pandemic challenged the scientific world and pharmaceutical industry to rapidly develop and implement controlled clinical trials (CCTs) testing for both repurposed and novel agents to treat the disease. Due to the rapidity of onset of the epidemic in China and Continental Europe, CCTs could not be rapidly activated during the initial phase of the pandemic (February–May 2020), but CCTs were promptly activated in the United Kingdom and the United States of America (USA), where the epidemic was delayed by several weeks [3–5].

Early in the pandemic, COVID-19 afflicted large urban centers to a greater degree where the density of the population and the travel traffic were more intense. The CCTs tended to be activated primarily in academic clinical centers in urban settings in areas with sizeable economic affluence. With the widespread of the epidemic/pandemic, COVID-19 cases increased rapidly in both urban and rural settings. Nevertheless, activation of CCTs has traditionally been poor at non-urban/rural centers, and therefore access to CCTs remained limited in the rural and underserved populations [6]. The implementation of CCTs in rural areas during a pandemic was faced with many challenges including a lack of resources at the patient care team level, the inexperience of patient care team members in CCT conductance and workflow interruption, and global inexperience with remote data monitoring [7]. We herein report our experience in activating a multicenter clinical trial at a rural satellite hospital during the COVID-19 pandemic using remote monitoring.

## Methods

### Clinical Setting

VCU Health Community Memorial Hospital (VCU-CMH) is a 70-bed community hospital located in South Hill, VA, USA, part of the larger VCU Health network, with the lead institution being VCU Health Medical College of Virginia Hospital (VCU-MCV). VCU-CMH primarily serves the rural and underserved population of southern Virginia and northern North Carolina, a geographical area that is classified as a healthcare shortage area by Health Resources and Service Administration (HRSA) [8]. VCU-CMH primarily serves the Mecklenburg, Lunenburg, and Brunswick counties in the state of Virginia attending a population of 100,000 inhabitants, which includes females (51.3%), persons aged 65 years or older (25.9%), Black (34%), and Hispanic or Latino or Ethnic minorities (4%) [9]. It includes an economically disadvantaged population with 20% persons below the poverty line with an annual household income of 20,000 dollars or less.

### Clinical Trial

We describe an example of a COVID-19 CCT activated at VCU-CMH entitled the Study of Efficacy and Safety of Canakinumab Treatment for cytokine release syndrome (CRS) in Participants with COVID-19-induced Pneumonia (CAN-COVID) trial (ClinicalTrials.gov Identifier: NCT04362813). We used the local resources at the facility and remote guidance and oversight from the VCU-MCV resources using a closed-loop communication network (i.e. shared electronic health records [EHRs], institutional review board [IRB], office-sponsored program). CAN-COVID is a Phase III, multicenter, randomized, double-blind, placebo-controlled study to assess the efficacy and safety of canakinumab in patients with COVID-19-induced pneumonia and CRS.

The study enrolled patients to canakinumab or placebo, in addition to standard-of-care (SOC) per local practice, which may include antiviral treatment, glucocorticoids, and/or supportive care. Patients were randomized in a 1:1 ratio to either canakinumab and SOC or placebo and SOC groups. The primary outcome was measured in terms of survival without requiring invasive mechanical ventilation from day 3 to day 29. Secondary outcomes were measures as COVID-19-related death rate, ratio to baseline in C-reactive protein (CRP), ratio to baseline in serum ferritin, and ratio to baseline in D-dimer during the 4 weeks after study treatment. The principal investigator monitored safety in the context of adverse events, serious adverse events , clinically significant laboratory changes, and vital signs for 127 days after the treatment. Dr. Antonio Abbate, MD, PhD, served as the principal investigator (PI) for the study at VCU Health.

### IRB Oversight

The study was submitted to the VCU IRB in Richmond, VA, for initial review and reviewed by the Advarra IRB. The IRB protocol listed both VCU-MCV and VCU-CMH as hospitals for screening, recruitment, and treatment.

### Research Team

The principal investigator and main research coordinator were located on the VCU-MCV Hospital campus, and they coordinated a team of investigators and coordinators locally. The team met virtually at least once daily. All members of the team had remote access to the shared EHR for VCU-MCV and VCU-CMH Hospitals. The Chief Medical Officer and Chief Nursing Officer at the VCU-CMH identified physicians and nurses who were qualified and willing to participate in the research team. The team members received training through videoconferencing from principal investigators, the coordinator, and the sponsor.

### Investigational Pharmacy

On this trial, VCU-CMH pharmacy functioned as a satellite with dispensing oversight and trial workup and ongoing review performed by VCU-MCV Investigational Drug Service. The investigational drug, canakinumab, was shipped from the sponsor to the VCU-MCV investigational pharmacy in Richmond, VA, and then from there to the investigational satellite pharmacy in South Hill, VA, in a styrofoam cooler with a min/max/average temperature monitor and fridge/freezer packs for initial supply. Subsequent shipments were sent directly from sponsor to VCU-CMH pharmacy. Designated unblinded pharmacists were identified at both locations. Once it was confirmed that a given consenting subject met study criteria and the PI had approved, the study team submitted patient information required for stratification and randomization. The study team followed with an electronic order for the investigational product (drug or placebo) that contained necessary information (subject screening number, patient weight, and dose if randomized to active treatment) into the EHR for pharmacist review and once approved and delivered, nurse charted. The unblinded pharmacy team located in the VCU-MCV Hospital was then notified of the patient’s treatment assignment by e-mail from the sponsor’s Interactive Web Response System (IWRS). Following review of both the electronic order and e-mail, a designated unblinded pharmacist at VCU-CMH prepared the investigational drug, documented the preparation procedures, and coordinated delivery to the treatment area for administration.

### Pathology

The study protocol included the use of a local pathology laboratory as well as a central laboratory. For the local pathology laboratory, most of the assays were performed onsite at VCU-CMH, whereas others (CRP and ferritin) were performed at the main laboratory at VCU-MCV.

### Coordination and Nursing

One principal clinical coordinator served as liaison with the sponsor. She communicated with the principal investigator multiple times daily, mostly virtually, and with the rest of the team on a regular basis. A regulatory coordinator assisted the principal coordinator and the principal investigator. The principal coordinator and the principal investigator were responsible for communicating with the study team at VCU-CMH. Research nurses from the Clinical Research Unit at VCU-MCV assisted with the training of a core group of nurses at VCU-CMH using in-person and virtual meetings.

### Screening

The principal investigator and a core group of co-investigators from VCU-MCV and VCU-CMH used the EHR at least once daily for potential candidates using a dedicated electronic platform in the EHR to track COVID-19 admissions at both institutions. Once a potential subject was identified at VCU-CMH, the principal investigator or one of the other team members reached out to the team onsite at VCU-CMH and initiated a discussion including also the clinical providers caring for the subject to verify inclusion and exclusion criteria.

### Recruitment and Consenting

After confirmation of inclusion and exclusion criteria, the subject was approached either by the principal investigator or another investigator at VCU-MCV using a telehealth videoconference device or by one of the investigators onsite at VCU-CMH in-person. A paper copy of the IRB informed consent form was provided to each subject by nurses. Communication between the research team and the subjects was often achieved using a secure connection on a tablet equipped with a video camera. After the subject had sufficient time to review the informed consent form, understand the content, have all questions answered, the subject was asked to sign the form and documentation of signature was obtained and maintained in an electronic file.

### Treatment

Investigational treatment was administered by a core group of adequately trained and supervised nurses at VCU-CMH. The nurses were instructed to monitor for clinical signs and symptoms and to document the administration of the drug in the EHR.

### Follow-up

Clinical data were extracted daily from the EHR by the clinical research team at VCU-MCV, and the principal investigator or another member of the team made periodic phone calls up to 127 days for follow-up as per protocol.

### Monitoring

The sponsor performed monitoring of the study. The monitors reviewed the data entered in the electronic case report forms (eCRFs) and they had access to the source documents through a limited-access EHR option and to other documents not in the EHR through protected share document options. All the monitoring was coordinated through the central clinical research coordinator in the VCU-MCV campus.

### Enrollment Results

The target enrollment was set for 40 subjects and we anticipated to have 40% of subjects to be of historically underpresented minorities. Enrollment at VCU-MCV was activated on May 8, 2020, and at VCU-CMH on May 10, 2020. By July 17, 2020, we had enrolled 40 subjects and we were approved to continue enrollment, and we ultimately enrolled 51 patients with COVID-19 ending August 13, 2020. Of the 51 subjects, 35 (69%) patients were enrolled at VCU-MCV and 16 (31%) at VCU-CMH. Among the patients enrolled at VCU-CMH, 37.5% were female, 62.5% Black, and had a median age of 60 (interquartile range 56 to 68) years (Table 1). The screen failure rate was 404 of 455 (89%): 17 of 58 (29%) at VCU-CMH and 34 of 360 (9.2%) at VCU-MCV.

**Table 1.** Clinical and demographic characteristics of patients enrolled in the CAN-COVID trial at VCU

Characteristic	VCU-MCV	VCU-CMH
*N*	31	16
Age, median (interquartile range), years	57 (49–66)	60 (56–68)
Males, *N* (%)	23 (65.5%)	10 (62.5%)
Race		
White, *N* (%)	18 (51.4%)	6 (37.5%)
Black, *N* (%)	17 (48.6%)	10 (62.5%)
Other, *N* (%)	0	0
Ethnicity		
Hispanic, *N* (%)	12 (34.3%)	0

CAN-COVID: Study of Efficacy and Safety of Canakinumab Treatment for CRS Participants with COVID-19-induced pneumonia. VCU-CMH: VCU Health Community Memorial Hospital. VCU-MCV: VCU Health Medical College of Virginia Hospital.

### Billing

Billing coordination was managed by the Clinical Research Unit on the VCU-MCV Hospital and facilitated by being within one single business entity and using a single EHR.

## Discussion

**Fig. 1.**
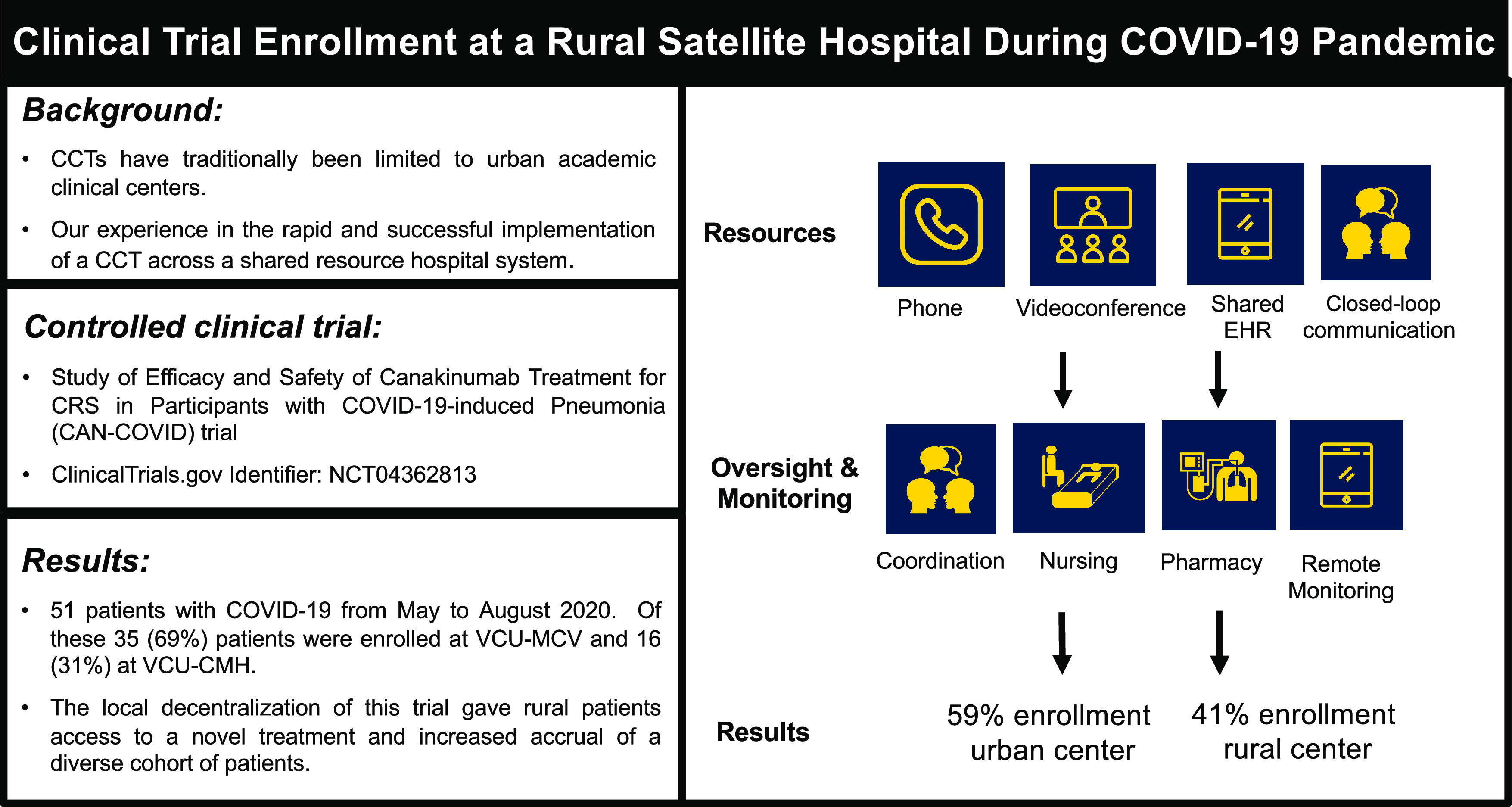
Schematic overlook of patient recruitment for the CAN-COVID trial at VCU-CMH. Due to the enormous spread of COVID-19, FDA released a guidance providing recommendations for remote/virtual assessments, including considerations for remote data collection. The possibility of remote working with clinical trials greatly helped decentralizing clinical trials that were highly needed due to the lack of effective drugs to treat COVID-19 patients. Legend. CAN-COVID: Study of Efficacy and Safety of Canakinumab Treatment for cytokine release syndrome (CRS) in Participants with COVID-19-induced Pneumonia. CCT: controlled clinical trial. COVID-19: coronavirus disease 2019. EHR: electronic health record. VCU-CMH: VCU Health Community Memorial Hospital. VCU-MCV: VCU Health Medical College of Virginia Hospital.

The COVID-19 pandemic has rapidly transformed how CCTs are conducted. Both the US Food and Drug Administration (FDA) and the European Medicines Agency (EMA) expediently issued guidance on changes during the pandemic to protect patients and facilitate continued execution of CCTs while maintaining good clinical practice standards [10,11]. Both of these guidance documents cover remote site monitoring, handling informed consent in remote settings, and the importance of preserving data integrity and audit trail. The FDA guidance provides detailed recommendations for remote/virtual assessments, including considerations for remote data collection. The distant operation of clinical trials has been proposed as a model to reduce participant burden and increased logistical demands [12]. The use of telemedicine and mobile healthcare providers, medical product supply chain, investigator delegation and oversight, and safety monitoring considerations has been increasingly advocated as a model to decentralized clinical trials [12]. COVID-19 has changed the medical community with a quick adaptation to telemedicine and telehealth. Healthcare systems have had a paradigm shift from office visits to telemedicine visits to limit travel and transmission of infections and to promote continuity of care [13]. Thus, herein, we shared our experience on how we utilized telemedicine, with an institutional oversight to execute a large randomized controlled multicenter trial in a rural satellite hospital (Fig. 1).

Telemedicine was essential in the start-up and execution of the trial. The study team member selection and training were completed remotely through teleconference. The screening process was completed through remote access in the EHR. The enrollment and recruitment were often completed through the use of videoconferencing with the subjects. Safety and outcome measures were captured through remote monitoring of the EHR. The coordination of the investigational treatment was performed remotely. The only in-person interventions were the preparation and administration of the investigation drug and the blood draws. This allowed for the experienced PI and team in the main site to coordinate the study and for a rather small team onsite to complete the study. There were no protocol violations at either site.

Remote execution of clinical trials can facilitate decentralization of clinical trials. It has a growing feasibility with the growth of technology, infrastructure, and easy adaptation of healthcare systems to telehealth models seen during the pandemic. Trial execution through telemedicine, local healthcare providers, and/or communication technologies is not bound by the geographic limitations that affect traditional trial models. Therefore, decentralized clinical trials can help recruit participants from anywhere beyond the geographical boundaries, potentially resulting in accelerated enrollment and more diverse participants’ representative of the target population. Moreover, follow-ups and measurements can be more frequent or even continuous because they are not restricted by scheduled clinic visits. A decentralized approach allows trial participants to take part in clinical research from anywhere, including the remote parts of country as seen in our experience. Decentralized clinical trial approaches may lessen participant burden (e.g. travel costs and time loss), which enhances retention and facilitates certain research that may otherwise be unduly burdensome using traditional models^8^. Expansion of a trial to a satellite facility in our experience was challenged by immature digital infrastructure, limited experience with the approach, and the perception of regulatory barriers. While these barriers were long-standing, the COVID-19 pandemic made them more clearly apparent and provided a sense of urgency.

Execution of CCTs in rural satellite facilities using remote monitoring can effectively decentralize clinical trials beyond the social, economic, and geographical barriers. Decentralized clinical trials can lead to accelerated enrollment of a more diverse participants representative of the target population. The screen failure rate in the satellite site was also favorably similar as compared with the urban site.

### Conclusion

We herein report our experience in the rapid and successful implementation of a CCT across a shared resource hospital system. The local decentralization of this trial gave rural patients access to a novel treatment option and across a time frame in the pandemic when zero to only two (dexamethasone and remdesivir) treatment options were recommended and enabled an increased accrual of a diverse cohort of patients.
